# A community partnership to evaluate the feasibility of addressing food insecurity among adult patients in an urban healthcare system

**DOI:** 10.1186/s40814-022-01013-3

**Published:** 2022-03-09

**Authors:** Katherine Scher, Aaron Sohaki, Amy Tang, Alexander Plum, Mackenzie Taylor, Christine Joseph

**Affiliations:** 1 Population Health Management and Clinical Coordination, Henry Ford Health System, Detroit, USA; 2 Population Health Management, Henry Ford Health System, Detroit, USA; 3 Department of Public Health Sciences, Henry Ford Health System, 1 Ford Place, 3E, Detroit, MI 48202 USA

**Keywords:** Food insecurity, Food banks, Electronic medical record, Healthcare utilization, Hunger vital sign

## Abstract

**Background:**

Food insecurity (FI) is a significant public health problem. Possible sequelae of prolonged food insecurity include kidney disease, obesity, and diabetes. Our objective was to assess the feasibility of a partnership between Henry Ford Health System (HFHS) and Gleaners Community Foodbank of Southeastern Michigan to implement and evaluate a food supplementation intervention initiated in a hospital outpatient clinic setting.

**Methods:**

We established a protocol for using the Hunger Vital Signs to screen HFHS internal medicine patients for food insecurity and established the data sharing infrastructure and agreements necessary for an HFHS-Gleaners partnership that would allow home delivery of food to consenting patients. We evaluated the food supplementation program using a quasi-experimental design and constructing a historical comparison group using the electronic medical record. Patients identified as food insecure through screening were enrolled in the program and received food supplementation twice per month for a total of 12 months, mostly by home delivery. The feasibility outcomes included successful clinic-based screening and enrollment and successful food delivery to consenting patients. Our evaluation compared healthcare utilization between the intervention and historical comparison group during a 12-month observation period using a difference-in-differences (DID) analysis.

**Results:**

Of 1691 patients screened, 353 patients (20.9%) met the criteria for FI, of which 340/353 (96.3%) consented, and 256/340 (75.3%) were matched and had data sufficient for analysis. Food deliveries were successfully made to 89.9% of participant households. At follow-up, the intervention group showed greater reductions in emergency department visits than the comparison group, −41.5% and −25.3% reduction, respectively. Similar results were observed for hospitalizations, −55.9% and −17.6% reduction for intervention and control groups, respectively. DID regression analysis also showed lower trends in ED visits and hospitalizations for the intervention group compared to the comparison group.

**Conclusions:**

Results suggest that community-health system partnerships to address patient-reported food insecurity are feasible and potentially could reduce healthcare utilization in these patients. A larger, randomized trial may be the next step in fully evaluating this intervention, perhaps with more outcomes (e.g., medication adherence), and additional covariates (e.g., housing insecurity and financial strain).

## Key messages regarding feasibility

- What uncertainties existed regarding the feasibility?

There is a paucity in the literature regarding the feasibility of collaborating with a community food bank to implement (identify, enroll, evaluate) a hospital-based food supplementation program for food-insecure adult patients.

- What are the key feasibility findings?

Implementation of a program to address food insecurity in adult patients is feasible; however, key components included consideration of clinic workflows regarding patient screening and enrollment, collaboration with a local community food bank, and the inclusion of home deliveries.

- What are the implications of the feasibility findings for the design of the main study?

A larger, more rigorous study may be needed to determine if food supplementation can improve health outcomes for food-insecure patients. Program content for a comparison group in a randomized trial of food supplementation will need to be carefully considered.

## Background

Food insecurity, a condition defined as “the disruption of food intake or eating patterns because of a lack of money and other resources,” has become a leading public health issue in the USA [1]. From 2014 to 2016, nearly 14.3% of households in Michigan reported food insecurity in the past 12 months, which is nearly double the desired Healthy People 2020 target of 6.0% [2]. The rate is much higher in the city of Detroit, with nearly 33% of households reporting food insecurity [3]. In 2017, nearly 30,000 individuals did not have access to the 74 full-line grocery stores located within Detroit city limits [4]. Full-line grocery stores are defined as stores that carry higher quality, fresh foods with a better selection and lower cost compared to smaller food stores. In 2015, Taylor and Ard reported that Detroiters travel twice as far to reach a full-line grocery store than they do to reach fast-food restaurants and convenience stores [5], although more recent reports suggest that other social and environmental factors may contribute more to food insecurity than proximity to stores, illustrating the complexity of these relationships [6].

Food insecurity has been shown to significantly impact health outcomes, especially for those who are socioeconomically disadvantaged [7, 8]. The most recent US estimates indicate that households experiencing poverty, and those of non-White ethnicity, are more likely to be food insecure [9, 10]. Individuals who identify as food insecure may use coping strategies such as postponing or foregoing medical care, rationing food, or purchasing low-cost, nutrient-poor foods in order to extend budgets [11]. Not only are these health-compromising coping strategies associated with food insecurity harmful, but they can also contribute to malnutrition, increased risk of poor health, and exacerbation of existing chronic conditions [12, 13]. Possible long-term negative physical and mental health sequelae arising from food insecurity include sleep disorders, kidney disease, obesity, diabetes, and depression [12, 13].

Significant increases in healthcare expenditures have been associated with food insecurity, which can be costly to healthcare systems [14]. Exacerbation of chronic conditions listed above can result in increased physician encounters and office visits, emergency room visits, hospitalizations, and expenditures for prescription medications [7]. Data from the Medical Expenditure Panel Survey and the National Health Interview Survey showed that healthcare system incremental costs for chronic disease in older patients was higher for those that were food insecure compared to those who did not meet the criteria for food insecure [14]. In 2014, the direct and indirect health-related costs of hunger and food insecurity in the USA were estimated to be approximately $160 billion [7]. In a retrospective cohort study that examined the relationship between food insecurity and healthcare expenditures, individuals with food insecurity reported approximately $1800 higher annual healthcare expenditures and were more likely to incur expenditures for inpatient hospitalizations and prescription medications than their food-secure counterparts [12].

Previous studies focusing on this topic have examined the prevalence of food insecurity, inequitable access to food sources among low-income populations, and the negative patient health outcomes associated with food insecurity [8, 10–12, 15]. To our knowledge, there are few interventions implemented in healthcare systems with the intent of reducing healthcare utilization, e.g., emergency department visits and hospitalizations [10, 11]. In one study, infant formula and other non-food resources were provided to food-insecure families attending a primary care clinic. Recipients of this help were more likely to undergo preventive care services, such as infant lead testing and developmental screening during the observation period [15]. In another study, home delivery of meals reduced emergency department and inpatient visits, as well as medical expenditures, in a sample of dually Medicare and Medicaid eligible adults [16]. We are unaware of interventions addressing food insecurity in general internal medicine clinics, with healthcare utilization as an outcome.

We describe the evaluation of a food supplementation intervention, Henry’s Groceries for Health, implemented at Henry Ford Health System (HFHS), a large, clinically integrated health system in southeast Michigan, with headquarters located in Detroit. The first objective of the Henry’s Groceries for Health program was to assess the feasibility of a health system partnership with a community organization in providing food to a targeted subgroup of patients. For this first objective, we sought to:

- Screen for food insecurity in a clinical setting, defining success as implementation of a clinic-based screening and referral protocol and reaching targeted enrollment.
- Develop infrastructure necessary for a health system — community organization partnership to supply food to patients meeting program criteria, defining success as establishing the necessary protocols and data agreements between HFHS and Gleaners to share the limited data necessary for food delivery to consenting patients.

A second objective was to demonstrate measurable improvement in patient health by addressing food insecurity through food supplementation in a primary care environment, defining success as conduct of the evaluation including collection of study outcomes and construction of a historical comparison group using the electronic health record (EHR). We hypothesized that supplemental food provided to persons screened as food insecure would result in decreases in healthcare utilization, specifically, emergency department visits and hospital admissions.

### Collaborators and setting

This project was the result of a collaboration between HFHS and Gleaners Community Foodbank of Southeastern Michigan (Gleaners). Gleaners, headquartered in Detroit, serves as a vital link between available food and those who need it most by providing nourishing food and nutrition education to households in metro Detroit and surrounding regions; operating distribution centers in Wayne, Oakland, Macomb, Livingston, and Monroe counties; and providing food to 528 partner soup kitchens, food pantries, shelters, and other agencies throughout southeast Michigan [17].

HFHS is a not-for-profit corporation based in Detroit, MI, comprising hospitals, medical centers, and a large group practice, the Henry Ford Medical Group (HFMG), with more than 1200 physicians practicing in over 40 specialties. HFHS owns Health Alliance Plan, a managed care organization serving southeast Michigan. The pilot program was implemented at three Henry HFMG primary care clinics located in Wayne County: two clinics were in the city of Detroit (pop. 674,841) and one clinic was in the city of Taylor (pop. 61,379), a small city located about 5 miles west of the southern border of Detroit and about 15 miles southwest of downtown Detroit [18].

## Methods

### Study design

**Fig. 1:**
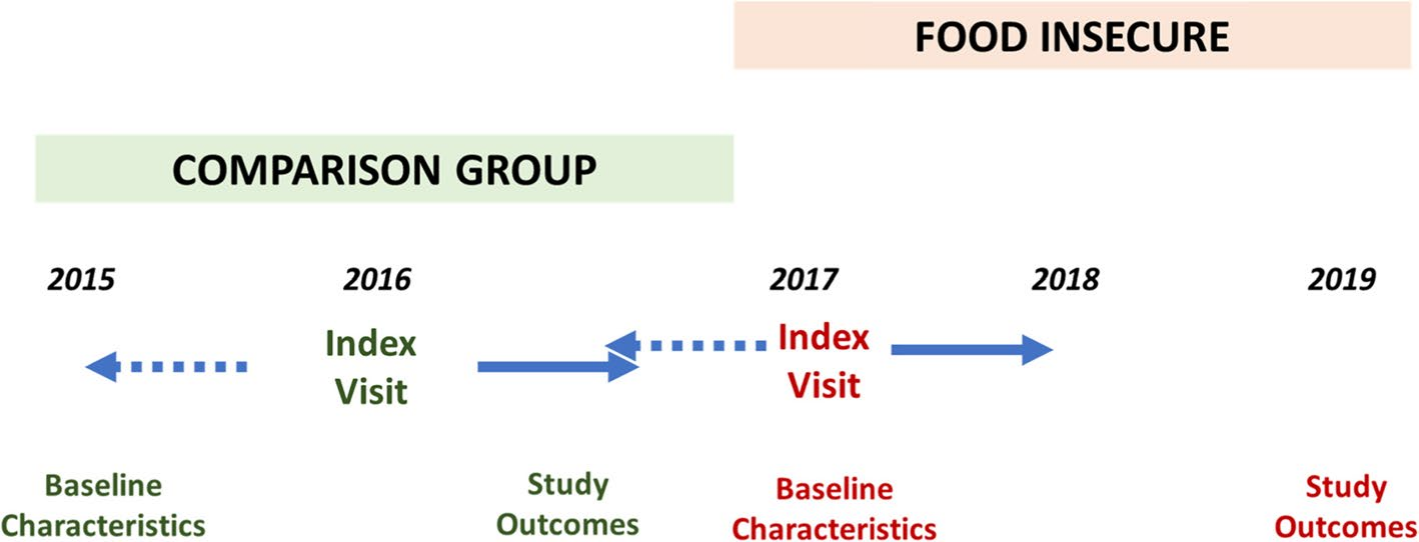
Study design with the historical EMR-derived comparison group

All aspects of this pilot study were approved by the Henry Ford Health System Institutional Review Board (IRB #11733). This was a quasi-experimental evaluation conducted among patients recruited from selected HFHS general internal medicine clinics. A historical comparison group was identified using the electronic medical record (EMR). An index visit was identified for each group (Fig. 1). For the intervention group, the index visit was that visit at which the patient was screened and consented for the study. For the historical control group, the index visit was the first encounter occurring between 11/1/2015 and 10/31/2016. Emergency department visits and hospitalizations occurring during the post-index 12-month follow-up period were retrieved using the EMR for both groups. To assign morbidity, we used ICD 9/10 codes for encounters occurring up to 12 months prior to the index visit.

### The Henry’s Groceries for Health intervention

The Henry’s Groceries for Health program was designed by the HFHS Population Health Management Department in collaboration with Gleaners and the HFHS Department of Public Health Sciences. Patients were screened for food insecurity and, upon meeting eligibility criteria and providing consent, were enrolled in the program. Enrolled patients received food supplementation twice per month for a total of 12 months, usually by home delivery, although some patients of the Henry Ford Health System Taylor Clinic traveled to the Gleaner’s Food Bank located approximately 3 miles away from the clinic. The program began with a starter package containing staples such as spices, grains, low-sodium canned goods, and cooking oils. Subsequent food packages, in alignment with the USDA My Plate recommendations, contained 45% fruits and vegetables and 15% protein, 15% grains, and 25% dairy [19]. Recipes, cooking tips, and food storage information were included with each food package. Participants received reminder calls for delivery the day before each scheduled delivery or pick-up date from an HFHS Population Health staff member, who also assessed the need for changes to package contents. Based on this information, future packages were adjusted or customized.

### Food scheduling and delivery

Patient-reported data for this study was collected and managed using REDCap, a HIPAA-compliant electronic data capture tool for collecting and managing study data [20, 21]. In addition to baseline and patient experience surveys, REDCap was used by both HFHS employees and Gleaners employees to securely communicate food delivery schedules. Gleaners employees had restricted access to REDCap reports and data collection features used to set up delivery schedules, make deliveries, and record outcomes for these activities. Participant information shared with Gleaners included consenting participant name, address, and phone number, in addition to food box preferences and delivery specifics.

### Intervention group

Patients were recruited at one of the three HFMG clinics from November 4, 2017, to May 11, 2018. Patients presenting to participating clinics were initially screened by clinic medical assistants; however, a preliminary assessment found that medical assistants objected to the length of the entire screening, eligibility, and enrollment process. It was determined that screening for food insecurity would need to be completed in two phases. Medical assistants would complete the screening (phase 1) and then refer the patient to a case manager who would complete the final portion of qualifying and enrolling the patient in the program (phase 2). Medical assistants used the Hunger Vital Signs to determine food insecurity [22]. This 2-item assessment is a validated screening tool for identifying households at risk for food insecurity using the following questions:

1. Within the past 12 months, we worried about whether our food would run out before we got money to buy more.
2. Within the past 12 months, the food we bought just did not last, and we did not have money to get more.

Answering affirmatively to at least one of the two questions was defined as “food insecure” for the purposes of this study and triggered an internal referral to an ambulatory case manager. To be included in the study, patients had to be 18 years of age or older, visiting one of the participating HFHS internal medicine clinics, and residing in metropolitan Detroit, which includes the city and surrounding areas including parts of the counties of Macomb, Oakland, and Wayne. Participants were excluded if they were less than 18 years of age, answered negatively to both questionnaire items, were unable to consent, had more than 3 persons in the household, and had one or more of selected conditions requiring restricted or special diets (i.e., dialysis or food allergies). The enrolling ambulatory case manager administered a baseline interview to eligible and consenting participants which included cultural/religious food preferences, possession of a refrigerator, oven, microwave, toaster oven, stove top, cooktop, electric skillet, or griddle. Patients were considered enrolled upon determination of eligibility and receipt of verbal informed consent by the ambulatory case manager. Patients meeting criteria for food insecurity but found to be ineligible were given information about alternative food resources, including referrals to local food pantries.

Within a week of being enrolled, ambulatory case managers called patients directly to schedule their deliveries, and within 24 h of delivery, Gleaners called to confirm the appointment time. Patients unable to receive delivery could reschedule. Within 48 h after delivery, ambulatory case management would call patients to confirm receipt of the food and ascertain satisfaction with the program, to date. This assessment included the following questions: “Did you find that your last food package met your needs?” “Did you eat all of the food you received?” “Did you use the recipes in the package?” and “Did anyone share the meals with you?” As additional social needs were raised by the patient, ambulatory case managers would respond in real time for follow-up and support.

### Historical comparison group

The historical comparison group was created using the HFHS electronic medical record (EMR). Initially, we identified all patients with at least 1 encounter occurring between 11/1/2015 and 10/31/2016 and who were 18 years of age or older at the time of the encounter. The first encounter identified within this time frame for each patient served as the index visit (Fig. 1). Using the index visit as a reference, we retrospectively collected “baseline” characteristics (demographics, healthcare utilization, morbidity) documented up to 12 months prior to the index visit date. These patients were matched to baseline characteristics of the intervention group using a combination of exact and propensity score matching on demographics, morbidity (encounters identified using ICD9 and ICD10 codes for asthma, hypertension, diabetes, coronary artery disease, congestive heart failure, COPD, and chronic kidney disease), and zip code of residence.

### Statistical methods

#### Propensity score matching for the historical comparison group

We used a combination of propensity score and exact matching to construct the matched historical comparison group and to evaluate and improve covariate balance between food insecure (intervention) and comparison groups. The propensity scores were estimated using a logit model with the full set of covariates, including age, marital status, asthma hypertension, diabetes, coronary artery disease, ED visits in the 12 months prior to the index visit, and food security status as the dependent variable, with a caliper of 0.25. Sex, race, and zip code were exactly matched.

#### Statistical analysis

Data are presented as means (standard deviations (sd)) for the continuous variables and as counts and percentages (*n*(%)) for the categorical variables. A two-sample *t* test or chi-squared test was used for the comparisons by food insecurity status (Table 1) and for comparisons between the food insecure and historical group (Table 2), as appropriate. Percentages of change were used to examine within-group reductions in the 12-month follow-up period with 95% confidence intervals. A confidence interval that did not include “1” was considered significant. Relative differences in ED visits between intervention and comparators before and after were estimated using the regression analysis of *difference-in-differences*. We tested for statistically significant differences in ED visits between intervention and comparison groups using an interaction term which gives the relative difference for the intervention (the change in use over time beyond the change observed in the comparison group (i.e., the “difference in differences”)).

**Table 1:** Characteristics of patients screened for food insecurity in three hospital outpatient clinics, November 4, 2017–May 11, 2018, and comparison by food insecurity status, *n*=1691

	Food insecurity				
Variable	Yes (***n***=353)		No (***n***=1338)		***p-***value
**Age, mean (sd)**	60.6	(13.7)	67.8	(16.3)	<0.001
**ED visits**^a^**, mean (sd)**	1.3	(2.2)	1.2	(2.9)	0.090
**Sex,*n*(%)**					0.003
**Female**	240	(68.0%)	791	(59.4%)	
**Male**	113	(32.0%)	540	(40.6%)	
**Race,*n*(%)**					<0.001
**Black**	304	(86.1%)	936	(70.3%)	
**White**	26	(7.4%)	289	(21.7%)	
**Other**	14^b^	(4.0%)	58^c^	(4.4%)	
**Decline**	9	(2.5%)	48	(3.6%)	
**Marital status,*n*(%)**					<0.001
**Married/partner**	79	(22.5%)	550	(41.3%)	
**Divorced/separated**	43	(12.3%)	151	(11.3%)	
**Widowed**	30	(8.5%)	185	(13.9%)	
**Single**	182	(51.9%)	417	(31.3%)	
**Other/unknown**	17	(4.8%)	28	(2.1%)	
**Morbidity**^d^**,*n*(%)**					
**Asthma**	37	(10.5%)	168	(12.6%)	0.289
**Hypertension**	271	(77.2%)	1020	(76.6%)	0.821
**Diabetes**	157	(44.7%)	577	(43.4%)	0.643
**Coronary artery disease**	62	(17.7%)	258	(19.4%)	0.465
**Congestive heart failure**	53	(15.1%)	292	(21.9%)	0.005
**COPD**	59	(16.8%)	194	(14.6%)	0.298
**Chronic kidney disease**	61	(17.4%)	409	(30.7%)	<0.001

^a^Emergency department visits in the 12-month pre-index visit; ^b^includes 6 self-report “Other”, 6 unknown, 1 Asian, and 1 listing more than 1 race; ^c^includes 26 self-report “Other”, 6 Asian, 20 unknown, 1 Am Indian, 1 Hispanic/Latinx, 1 Middle Eastern/North African, 1 listing more than 1 race, and 2 Native Hawaiian/Pacific Islander; ^d^ICD9 and 10 codes used: asthma: J45.20, J45.21, J45.22, J45.30, J45.31, J45.32, J45.40, J45.41, J45.42, J45.50, J45.51, J45.52, J45.901, J45.902, J45.909, J45.990, J45.991, J45.998; hypertension: I10; diabetes: E10.10, E10.11, E10.21, E10.22, E10.29, E10.311, E10.319, E10.321, E10.329, E10.331, E10.339, E10.341, E10.349, E10.351, E10.359, E10.36, E10.39, E10.40, E10.41, E10.42, E10.43, E10.44, E10.49, E10.51, E10.52, E10.59, E10.610, E10.618, E10.620, E10.621, E10.622, E10.628, E10.630, E10.638, E10.641, E10.649, E10.65, E10.69, E10.8, E10.9, E11.00, E11.01, E11.21, E11.22, E11.29, E11.311, E11.319, E11.321, E11.329, E11.331, E11.339, E11.341, E11.349, E11.351, E11.359, E11.36, E11.39, E11.40, E11.41, E11.42, E11.43, E11.44, E11.49, E11.51, E11.52, E11.59, E11.610, E11.618, E11.620, E11.621, E11.622, E11.628, E11.630, E11.638, E11.641, E11.649, E11.65, E11.69, E11.8, E11.9, E13.00, E13.01, E13.10, E13.11, E13.21, E13.22, E13.29, E13.311, E13.319, E13.321, E13.329, E13.331, E13.339, E13.341, E13.349, E13.351, E13.359, E13.36, E13.39, E13.40, E13.41, E13.42, E13.43, E13.44, E13.49, E13.51, E13.52, E13.59, E13.610, E13.618, E13.620, E13.621, E13.622, E13.628, E13.630, E13.638, E13.641, E13.649, E13.65, E13.69, E13.8, E13.9; coronary artery disease: I20.0, I20.1, I20.8, I20.9, I21.09, I21.19, I21.29, I21.3, I21.4, I24.0, I24.8, I25.10, I25.2, I25.5, I25.810, I25.811, I25.812, I25.89, I25.9, Z95.1, Z98.61; congestive heart failure: I11.0, I13.0, I13.2, I50.1, I50.20, I50.21, I50.22, I50.23, I50.30, I50.31, I50.32, I50.40, I50.41, I50.42, I50.43, I50.9; chronic obstructive pulmonary disease: J44.0, J44.1, J44.9; chronic kidney disease: N18.1, N18.2, N18.3, N18.4, N18.5, N18.6

**Table 2:** Characteristics of the study sample and matching results using exact match and propensity scores

Variable	Food insecure (***n***=256)		Historical comparison group (***n***=256)		***p-***value
**Demographics and marital status**					
**Age, mean (sd)**	60.3	(12.8)	61.0	(14.7)	0.62
**Female**^a^**,*n*(%)**	168	(65.6)	168	(65.6)	0.99
**African-American**^a^**,*n*(%)**	221	(86.3)	221	(86.3)	0.99
**Married,*n*(%)**	68	(26.6)	65	(25.4)	0.76
**Morbidity**					
**Asthma,*n*(%)**	29	(11.3)	39	(13.9)	0.99
**Hypertension,*n*(%)**	200	(78.1)	200	(78.1)	0.99
**Diabetes,*n*(%)**	116	(45.3)	112	(43.8)	0.72
**Coronary artery disease,*n*(%)**	46	(18.0)	46	(18.0)	0.99
**Selected healthcare utilization**					
**ED visits in the prior year, mean (sd)**	1.86	(3.66)	1.34	(2.46)	0.16
**ED visit category,*n*(%)**					
**0**	133	(52.0)	143	(55.9)	0.62
**1**	26	(10.2)	28	(10.9)	
**2**	37	(14.5)	37	(14.5)	
**>3**	60	(23.4)	48	(18.8)	

^a^Sex, race, and zip code of residence used for an exact match. All other variable distributions were constructed in the comparison group using propensity scores

#### Patient satisfaction

The selected questions from the post-delivery patient satisfaction survey were tabulated and described using percentages.

## Results

### Objective 1: Screen for food insecurity in a clinical setting

**Fig. 2:**
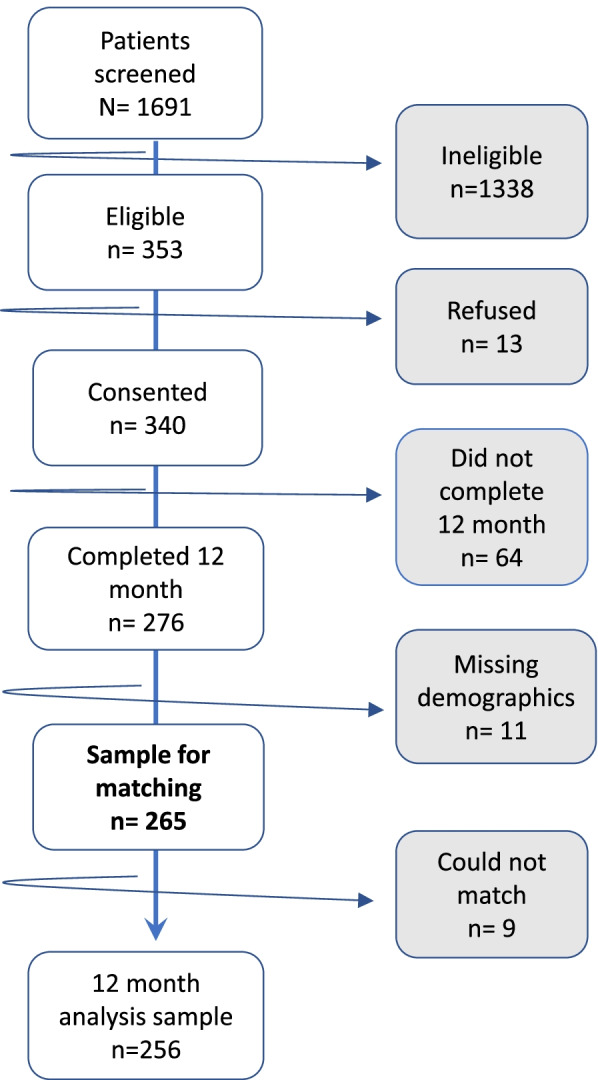
Breakdown of the study population

The first objective of the pilot was to address the feasibility of key operational details. Medical assistants conducted the screening and patients screened as food insecure were referred to the case manager. As noted above, success required screening in two phases: medical assistant referral to a case manager. This method allowed screening in the clinic setting. Recruitment and enrollment dispositions are presented in Fig. 2. A total of 1691 patients were screened for food insecurity in the clinic during the recruitment period, with 353 patients (20.8%) meeting the criteria for food insecurity.

### Objective 2: Successfully share data with our community partner maintaining adherence to HIPPA and other guidelines and conduct food deliveries

The use of REDCap by both HFHS and Gleaners allowed secure communication of food delivery schedules. Of the 353 patients with food insecurity, 340 (96.3%) consented to be in the study, of which 97% elected to have food delivered to their home. A total of 6519 deliveries were scheduled of which 5860 (89.9%) were “successful” (i.e., the enrolled participant received the food).

### Objective 3: Implement a quasi-experimental design to assess food supplementation impact on patient outcomes

Among all patients screened, we compared the characteristics of the food insecure (*n*=353) to those not meeting the criteria for food insecurity (*n*=1338) (Table 1). Food-insecure (FI) patients were significantly younger and more likely to be African American, female, and single. FI patients were less likely to have congestive heart failure and chronic kidney disease (Table 1). Of 340 consenting, 276/340 (81.2%) completed the 12-month survey. Of these, 256/276 (92.7%) had sufficient data for matching (Fig. 2). Characteristics of the study sample, variables used for matching, and prevalence of chronic diseases in the sample, as determined by encounters with corresponding ICD9/10 codes, are shown in Table 2. We did not observe any statistically significant differences in demographics, co-morbidities, and utilization for intervention patients meeting criteria for food insecurity and a historical control group obtained from the medical record.

We calculated the relative and absolute reduction within-group for emergency department visits and for hospitalizations as shown in Table 3. At the 12-month post-index visit, *n*=123 patients in the intervention group made 279 ED visits, representing a −41.5% relative reduction from baseline and per person average reduction was 0.77 (95% *CI* 0.40–1.15) The historical comparison group also showed significant relative reductions in ED visits (−25.3%) and per person reduction 0.34 (0.09–0.59) and while smaller in magnitude compared to the intervention group, the reduction was also significant. A similar trend was observed for hospitalizations (Table 3). A relative reduction of −55.9% and a significant per person reduction of 0.15 (0.01–0.29) in hospitalizations were observed for the intervention group, compared to a −17.6% relative reduction and −0.004 (−0.06–0.07) per person reduction in hospitalizations for the historical comparison group that did not reach statistical significance.

**Table 3:** Relative reduction of emergency department (ED) visits and hospitalizations for intervention (food insecure) and historical comparison groups

	(Baseline) 12-month pre-index visit		12-month post-index visit		Relative reduction	Average per person reduction (95% ***CI***)
	Visits	Patients	Visits	Patients		
**ED visits**						
Intervention group	477	*n*=123	279	*n*=122	−41.5%	0.77 (0.40–1.15)
Comparison group	344	*n*=113	257	*n*=90	−25.3%	0.34 (0.09–0.59)
**Hospitalizations**						
Intervention group	68	*n*=30	30	*n*=26	−55.9%	0.15 (−0.01–0.29)
Comparison group	34	*n*=27	28	*n*=24	−17.6%	−0.004 (−0.06, 0.07)

**Fig. 3:**
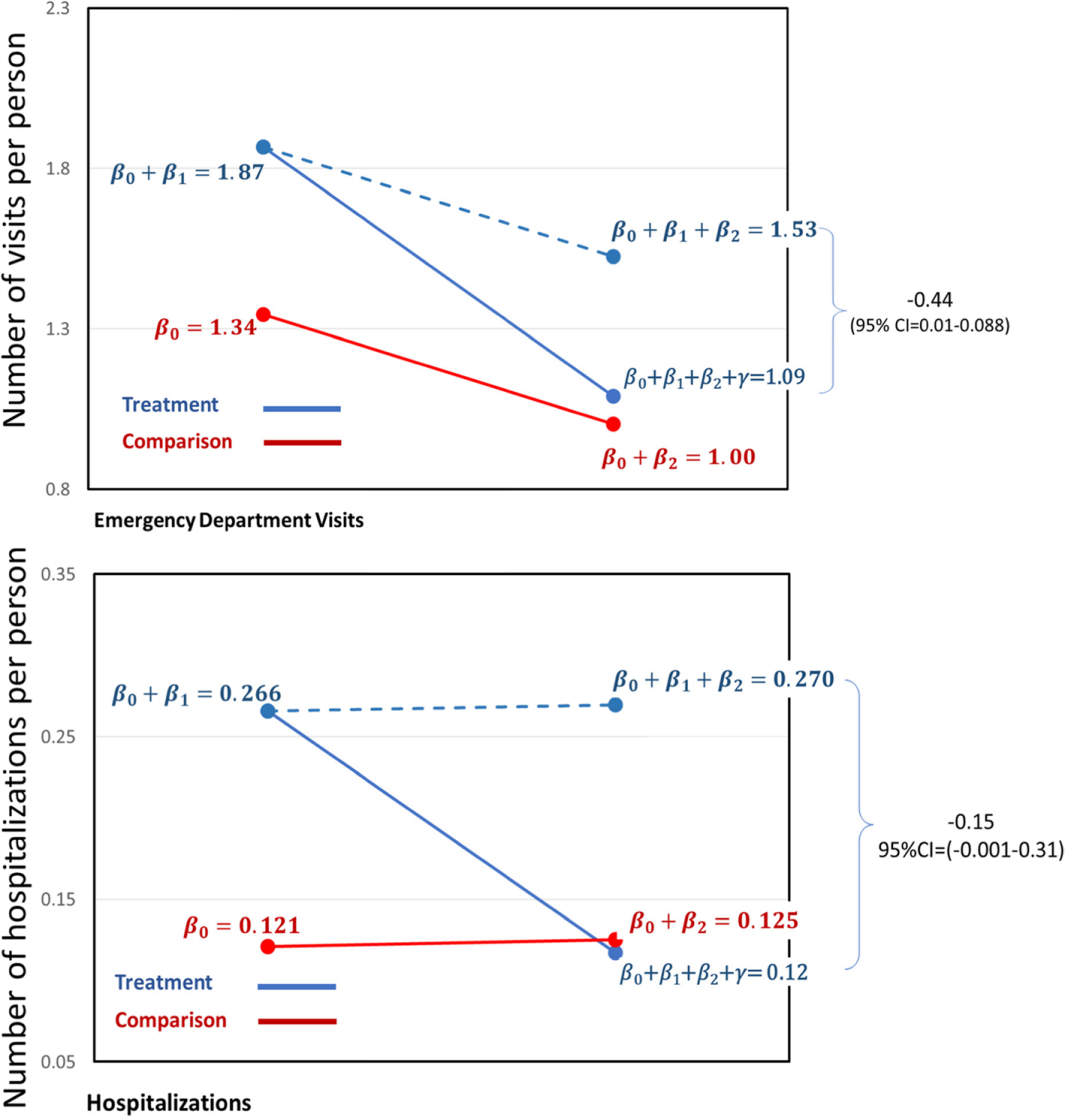
Results of difference-in-difference regression analysis for the 12-month follow-up period

Figure 3 shows the results of difference-in-differences regression analysis to determine if the trend in ED visits over time was the same for the intervention group compared to that of the historical comparison group. The trend in ED visits for 12 months for the intervention group was 0.44 (−0.01–0.88) visits/patient lower than that of the historical comparison group. Similarly, the trend for hospitalizations for the intervention group was 0.15 (−0.001–0.31) visits/patient lower than that of the historical comparison group.

### Patient satisfaction survey

A total of 260 patients (76.4% of the 276 that completed a 12-month follow-up survey) completed 1987 post-delivery surveys, averaging 7.64 surveys/participant. According to the aggregated participant surveys, 95.8% reported the food met their needs, 90.7% of participants reported eating all the food, 50.3% reported using the dietary tips and recipes included with the food boxes, and 36.5% reported sharing the meals with others.

## Discussion

We piloted a food supplementation program in conjunction with Gleaner’s community food bank. In our considerations for addressing food insecurity, we elected to partner with Gleaner’s to conduct a small feasibility study to explore the effect of food supplementation on selected patient outcomes within a 12-month period. At the time of this feasibility study, existing evidence of success among healthcare systems conducting this type of project was limited. At the time, conducting a health system-wide randomized controlled trial seemed premature. We examined the (a) feasibility of screening in a clinical setting, (b) of sharing information necessary to conduct a pilot with our community partner and successfully delivering food packages to enrolled patients, and (c) of conducting an evaluation with a comparison group constructed using information obtained from the EMR and employed an analytic approach suitable for instances when randomization on an individual patient level is not possible [23]. In our analysis, we observed decreased ED and inpatient visits in a 12-month period among adult patients meeting criteria for FI and enrolled in the pilot.

Using the Hunger Vital Signs which consists of only two questions, we attempted to create operational efficiency that did not intrude too significantly on the workload of medical assistants, while still creating a warm handoff between the patient and a case manager who completed enrollment. In today’s busy clinics where staff are fully stretched, a streamlined approach is essential. Another option that could be considered is to focus on patients already in case management. This also has pros and cons. For example, this option may place emphasis on the sickest patients who are assigned to a case manager but may miss patients whose condition does not yet warrant a case manager but could worsen due to food insecurity [24, 25].

Sharing protected patient health information with community-based, social service providers like Gleaners has presaged the importance to healthcare systems of partnerships that facilitate the exchange of information to meet complex needs. Sometimes referred to as “community health information exchanges” [26], our partnership with Gleaners represented an analogue version of what we hope will grow into a sophistical, interoperable system of data exchange that ensures whole person care. In order to provide delivery through an external vendor like Gleaners, we were required to carefully consider HIPAA rules, as sharing patient addresses constitutes an exchange of protected health information. This was addressed by providing information in the informed consent with patients. Detroit is a city of few transit options and since transportation remains a significant barrier to accessing medical care, we surmised it would be a barrier to accessing other social services and goods [27]. While addressing food insecurity has been tried before with food pharmacies and food stands, these intervention designs typically do not include food delivery [28]. This intervention design targeted a core issue of food insecurity without inadvertently penalizing transportation insecure patients.

The second objective was to assess intervention impact on healthcare utilization. There is yet little information on how interventions that address food insecurity can impact health outcomes, specifically healthcare utilization. Of the two other interventions addressing food insecurity in healthcare settings mentioned in the introduction, Berkowitz et al. is the most similar to our study [12, 13, 16]. The Berkowitz intervention delivered medically tailored and untailored meals to a sample of dual-eligible (Medicare and Medicaid) patients and sought to reduce ED and inpatient visits as well as medical costs [16]. Investigators used concurrent non-participants as matched controls. Medically tailored meals were associated with fewer ED and inpatient visits and lower medical costs at the end of the observation period. Non-tailored meal delivery was associated with fewer ED visits, but the magnitude of effect was smaller, and the cost savings was $10 versus $200 for the tailored group [16]. Based on the results of the Berkowitz study, the addition of medically tailored meals, while another layer of complexity, may be worth the considerable cost savings [16].

There are limitations to this study. Our experience revealed important lessons around intervention design and patient engagement. To our knowledge, the evidence to support a randomized trial of a health system-derived food supplementation intervention had not yet clearly emerged in the literature at the onset of our project. The quasi-experimental design and use of a historical comparison group lend itself to inherent biases, the most important and obvious being that while our comparison group was selected based on the distribution of characteristics in the intervention group, patients in the comparison group were not screened for food insecurity. Differences in the proportion of food insecurity in the comparison group could bias study results, especially if factors related to food insecurity are also related to ED visits and hospitalizations. Other limitations relate to the assumptions operating in the difference-in-difference analysis. For example, our results assume that there are no time-varying differences between the intervention and comparison groups, which is a broad assumption as these groups have the same length of follow-up but are observed over a different period (i.e., index dates occurring 2016 and 2017–2018 for historical control and intervention groups, respectively). There is also an assumption that trends prior to the onset of the intervention (or prior to the index visit for the historical control group) are not significantly different. Attempts to fulfill this assumption were conducted through propensity score and exact matching. Propensity score matching can help to reduce bias; however, in this case, using this method also led to excluding 3.3% of patients (*n*=9) in our intervention group that could not be matched. These biases inherent in our study design and analysis may limit the generalizability of our findings. Finally, our data provide no conclusions about the mechanism by which food supplementation leads to fewer ED visits and hospitalizations. The association of food insecurity to healthcare utilization, however, has been shown in a previous longitudinal study using national data [29], and other studies have found that hypoglycemia and glycemic control were associated with more frequent ED visits among patients with diabetes [24, 30]. In this study, congestive heart failure, coronary artery disease, and chronic kidney disease were not more prevalent among patients reporting food insecurity, although, similar to diabetes, management of these conditions is often diet dependent and has been found to be associated with food insecurity [25, 31].

Despite the above limitations, results suggest that community-health system partnerships to address patient-reported food insecurity is feasible and potentially could reduce ED and hospitalizations in these patients. Home delivery is essential. A randomized trial in a larger study sample could be the next step in fully evaluating these issues. Randomizing patients found to be food insecure presents some ethical concerns in terms of a control group. Other studies have provided food vouchers or cash to the comparison group. A randomized trial could examine additional outcomes to support or inform observed patterns in utilization (e.g., medication adherence) as could the addition of covariates such as housing insecurity and financial strain.

## Data Availability

The datasets used and/or analyzed during the current study are available from the corresponding author on reasonable request.

## Abbreviations

DID: Difference-in-differences
ED: Emergency department
EMR: Electronic medical record
FI: Food insecure/food insecurity
HFHS: Henry Ford Health System
HFMG: Henry Ford Medical Group
IRB: Institutional Review Board
